# Supplementary material for: Perceived Access to Health Care Services and Relevance of Telemedicine during the COVID-19 Pandemic in Germany
Source: Int J Environ Res Public Health. 2021 Jul 19;18(14):7661. doi: 10.3390/ijerph18147661 (PMC8303178; doi:10.3390/ijerph18147661)
Supplement: Supplementary file 1 [file ijerph-18-07661-s001.zip › ijerph-1268409-supplementary.pdf]

Supplement Materials

Perceived Access to Health Care Services and Relevance of Telemedicine during the COVID-19 Pandemic in Germany

Lukas Reitzle [1,*], Christian Schmidt [1], Francesca Färber [1], Lena Huebl [2,3], Lothar Heinz Wieler [4], Thomas Ziese [1] and Christin Heidemann [1]

[1] Department of Epidemiology and Health Monitoring, Robert Koch Institute, 12101 Berlin, Germany; SchmidtChri@rki.de (C.S.); FaerberF@rki.de (F.F.); ZieseT@rki.de (T.Z.); HeidemannC@rki.de (C.H.)
[2] Department of Tropical Medicine, Bernhard Nocht Institute for Tropical Medicine, University Medical Center Hamburg-Eppendorf, 20359 Hamburg, Germany; l.huebl@uke.de
[3] I. Department of Medicine, University Medical Center Hamburg-Eppendorf, 20251 Hamburg, Germany
[4] Robert Koch Institute, 13353 Berlin, Germany; WielerLH@rki.de
* Correspondence: reitzlel@rki.de

Figure S1. Forest plot of logistic regression model for perceived availability of medical services (0 = no, not available; 1 = yes, available)

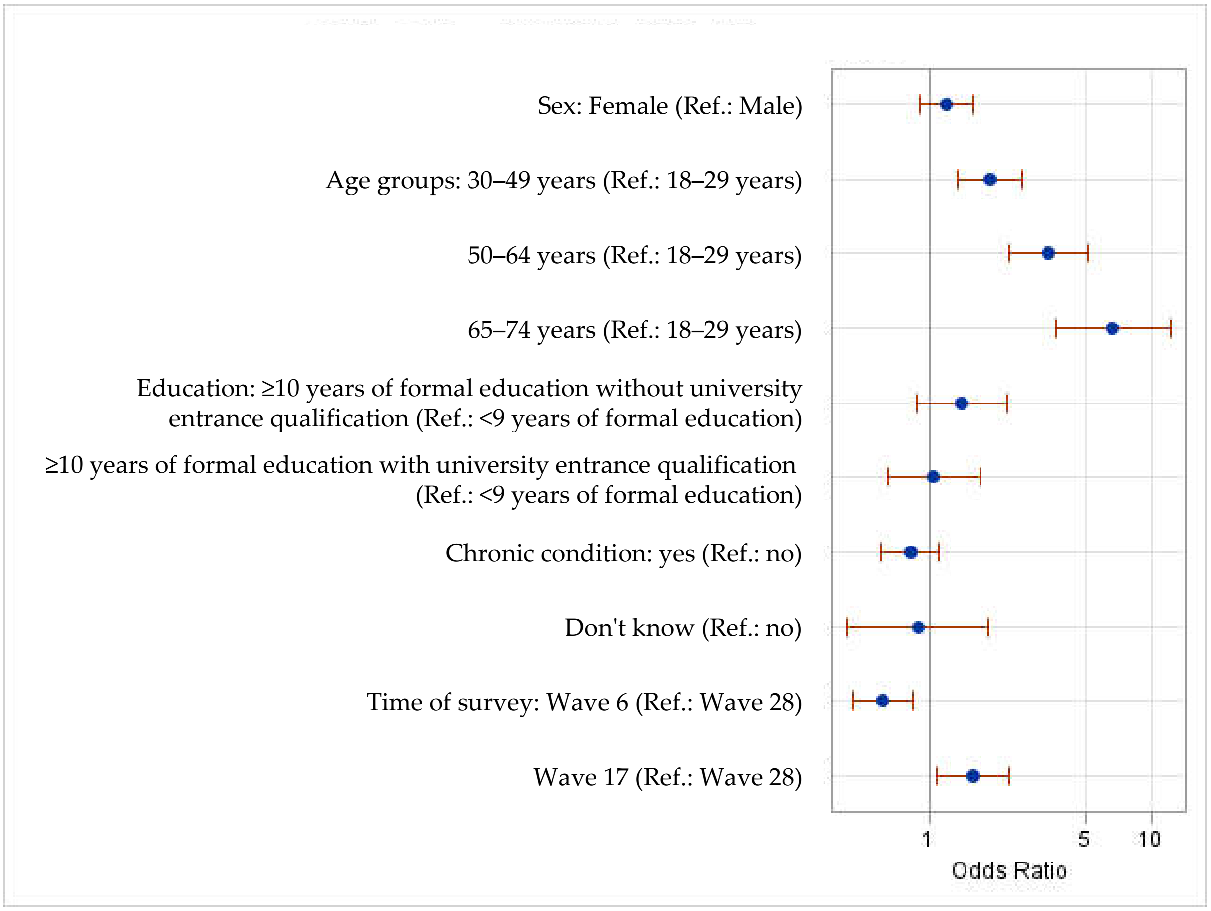

Table S1. Composition of study population compared to the German population regarding age and sex

	Wave 6 April 2020 (*n* = 773)	Wave 17 July 2020 (*n* = 941)	Wave 28 December 2020 (*n* = 868)	All three waves	German Population*
	n (%)	*n* (%)	*n* (%)	*n* (%)	*n* (%)
Male					
18–29 years	58 (14.9)	90 (19.8)	72 (17.4)	220 (17.5)	5,781,592 (19.5)
30–49 years	147 (37.9)	151 (33.2)	157 (37.9)	455 (36.2)	11,520,095 (38.9)
50–64 years	115 (29.6)	129 (28.4)	120 (29.0)	364 (29.0)	8,081,342 (27.3)
65–74 years	68 (17.5)	85 (18.7)	65 (15.7)	218 (17.3)	4,246,483 (14.3)
Female					
18–29 years	69 (17.9)	87 (17.9)	82 (18.1)	238 (18.0)	5,611,399 (18.7)
30–49 years	148 (38.4)	185 (38.1)	169 (37.2)	502 (37.9)	11,325,577 (37.8)
50–64 years	104 (27.0)	137 (28.2)	130 (28.6)	371 (28.0)	8,247,217 (27.5)
65–74 years	64 (16.6)	77 (15.8)	73 (16.1)	214 (16.2)	4,788,107 (16.0)

* German Population based on data from the 2011 census [1]

Table S2. Parameter estimates of logistic regression model for perceived availability of medical services (0 = no, not available; 1 = yes, available)

Parameter	Estimate	Standard error	*p*-value
Intercept	1.3686	0.2802	< 0.0001
Sex			
Male	Ref		
Female	0.1801	0.1403	0.1993
Age groups			
18–29 years	Ref		
30–49 years	0.6254	0.1718	0.0003
50–64 years	1.2216	0.2105	< 0.0001
65–74 years	1.8942	0.3048	< 0.0001
Education			
≤9 years of formal education	Ref		
≥10 years of formal education without university entrance qualification	0.3338	0.2399	0.1641
≥10 years of formal education with university entrance qualification	0.0497	0.2453	0.8396
Chronic condition			
No	Ref		
Yes	-0.1985	0.1524	0.1927
Don't know	-0.1196	0.3722	0.7480
Time of survey			
Wave 6 – April 2020	-0.4848	0.1631	0.0030
Wave 17 – July 2020	0.4510	0.1857	0.0152
Wave 28 – December 2020	Ref		

Table S3. Proportion of utilization of telemedicine among participants requiring medical appointments stratified by sex, age, education, chronic condition, and perceived availability of medical appointments (wave 28, *n* = 868)

	**Telephone consultation**		**Video consultation**		**No**		**Not needed**	
	% (95%-CI)	*n*	% (95%-CI)	*n*	% (95%-CI)	*n*	% (95%-CI)	n
Overall	15.4 (13.2–18.0)	134	7.6 (6.0–9.6)	66	63.7 (60.4–66.9)	553	20.6 (18.1–23.4)	179
Sex								
Male	19.8 (16.2–23.9)	82	10.4 (7.8–13.7)	43	59.9 (55.1–64.5)	248	20.0 (16.5–24.2)	83
Female	11.5 (8.8–14.7)	52	5.1 (3.4–7.5)	23	67.2 (62.7–71.4)	305	21.1 (17.6–25.2)	96
Age groups								
18–29 years	17.5 (12.3–24.4)	27	15.6 (10.7–22.2)	24	53.2 (45.3–61.0)	82	24.7 (18.5–32.1)	38
30–49 years	17.8 (14.0–22.3)	58	8.9 (6.2–12.5)	29	60.1 (54.7–65.3)	196	23.0 (18.7–27.9)	75
50–64 years	10.8 (7.5–15.3)	27	4.0 (2.2–7.3)	10	71.2 (65.3–76.5)	178	18.0 (13.7–23.3)	45
65–74 years	15.9 (10.7–23.0)	22	2.2 (0.7–6.5)	3	70.3 (62.1–77.3)	97	15.2 (10.1–22.2)	21
Education								
≤9 years of formal education	12.4 (7.3–20.2)	13	2.9 (0.9–8.5)	3	72.4 (63.1–80.1)	76	22.9 (15.8–31.9)	24
≥10 years of formal education without university entrance qualification	15.2 (11.5–19.8)	44	6.2 (3.9–9.6)	18	67.6 (62.0–72.7)	196	17.2 (13.3–22.0)	50
≥10 years of formal education with university entrance qualification	16.3 (13.2–19.9)	77	9.5 (7.2–12.5)	45	59.4 (54.9–63.8)	281	22.2 (18.7–26.2)	105
Chronic condition								
Yes	14.0 (10.6–18.3)	45	5.6 (3.6–8.7)	18	73.2 (68.1–77.8)	235	11.5 (8.5–15.5)	37
No	16.6 (13.6–20.0)	86	9.1 (6.9–11.9)	47	57.4 (53.1–61.6)	298	26.6 (23.0–30.6)	138
Don’t know	10.7 (3.5–28.5)	3	3.6 (0.5–21.5)	1	71.4 (52.4–85.0)	20	14.3 (5.5–32.5)	4
Access to medical appointments ensured								
Yes	15.0 (12.7–17.7)	119	6.1 (4.6–8.0)	48	64.9 (61.5–68.2)	514	21.5 (18.7–24.5)	170
No	19.7 (12.3–30.2)	15	23.7 (15.4–34.5)	18	51.3 (40.2–62.3)	39	11.8 (6.3–21.2)	9

95%-CI: 95% Confidence interval

References

1. Federal Statistical Office. Population (2011 Census): Germany, reference date, sex, age groups. Table 12111-0004. Available online: https://www-genesis.destatis.de/genesis//online?operation=table&code=12111-0004 (accessed on 14 July 2021).
